# (2*E*,5*E*)-2,5-Difurfurylidenecyclo­penta­none

**DOI:** 10.1107/S1600536809047278

**Published:** 2009-11-14

**Authors:** Shi-Ying Ma, Ze-Bao Zheng

**Affiliations:** a Department of Chemistry and Environmental Science, Taishan University, 271021 Taian, Shandong, People’s Republic of China

## Abstract

In the title mol­ecule, C_15_H_12_O_3_, the three five-membered rings are nearly coplanar: the dihedral angles between the cyclopentanone ring and the furan rings are 3.5 (2) and 9.7 (2)°, and the two furan rings form a dihedral angle of 7.2 (2)°. In the crystal structure, weak inter­molecular C—H⋯O hydrogen bonds help to consolidate the crystal packing.

## Related literature

For background to the use of bis­(aryl­methyl­idene)cyclo­alkanones as building blocks for the synthesis of biologically active heterocycles, see Guilford *et al.* (1999 ▶). For related structures, see: Du *et al.* (2007 ▶); Sun & Cui (2007 ▶); Wei *et al.* (2008 ▶).
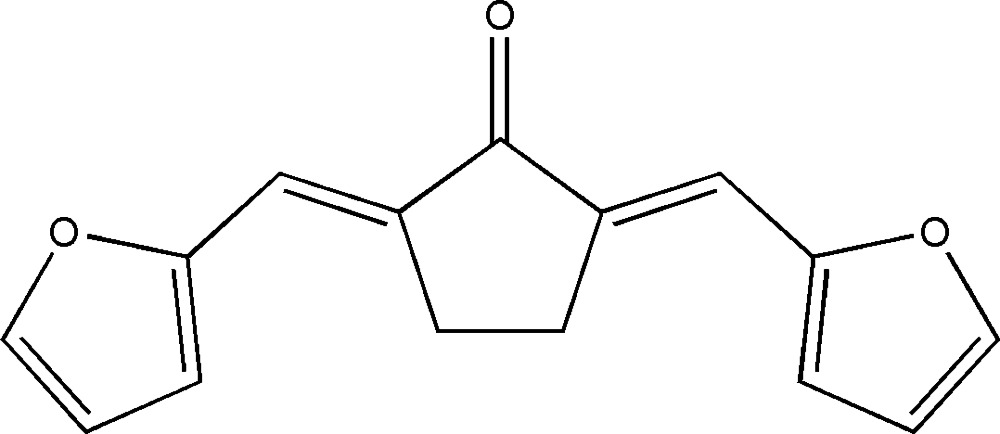


## Experimental

### 

#### Crystal data

C_15_H_12_O_3_
*M* *_r_* = 240.25
Monoclinic, 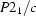

*a* = 5.9280 (9) Å
*b* = 8.5031 (13) Å
*c* = 23.280 (3) Å
β = 92.139 (3)°
*V* = 1172.6 (3) Å^3^
*Z* = 4
Mo *K*α radiation
μ = 0.10 mm^−1^
*T* = 298 K
0.12 × 0.08 × 0.05 mm

#### Data collection

Bruker SMART CCD area-detector diffractometer
Absorption correction: multi-scan (*SADABS*; Sheldrick, 1996 ▶) *T*_min_ = 0.989, *T*_max_ = 0.995
5793 measured reflections
2076 independent reflections
1083 reflections with *I* > 2σ(*I*)
*R*_int_ = 0.059

#### Refinement

*R*[*F* ^2^ > 2σ(*F* ^2^)] = 0.050
*wR*(*F* ^2^) = 0.129
*S* = 0.99
2076 reflections
164 parameters
H-atom parameters constrained
Δρ_max_ = 0.17 e Å^−3^
Δρ_min_ = −0.16 e Å^−3^

### 

Data collection: *SMART* (Siemens, 1996 ▶); cell refinement: *SAINT* (Siemens, 1996 ▶); data reduction: *SAINT*; program(s) used to solve structure: *SHELXS97* (Sheldrick, 2008 ▶); program(s) used to refine structure: *SHELXL97* (Sheldrick, 2008 ▶); molecular graphics: *SHELXTL* (Sheldrick, 2008 ▶); software used to prepare material for publication: *SHELXTL*.

## Supplementary Material

Crystal structure: contains datablocks global, I. DOI: 10.1107/S1600536809047278/cv2656sup1.cif

Structure factors: contains datablocks I. DOI: 10.1107/S1600536809047278/cv2656Isup2.hkl

Additional supplementary materials:  crystallographic information; 3D view; checkCIF report

## Figures and Tables

**Table 1:** Hydrogen-bond geometry (Å, °)

*D*—H⋯*A*	*D*—H	H⋯*A*	*D*⋯*A*	*D*—H⋯*A*
C15—H15⋯O1^i^	0.93	2.49	3.221 (4)	136
C4—H4*A*⋯O1^ii^	0.97	2.45	3.393 (3)	165

Symmetry codes: (i) 
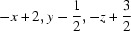
; (ii) 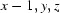.

**Fig. 1.**
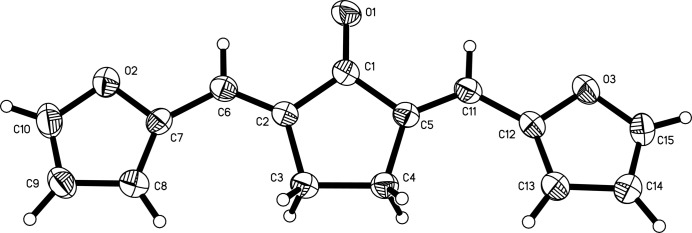
The molecular structure of (I), with displacement ellipsoids drawn at the 30% probability level.

## supplementary crystallographic information

### Comment

Bis(arylmethylidene)cycloalkanones are widely used as building blocks for the synthesis of biologically active heterocycles (Guilford *et al.*, 1999). In the present paper, we describe the crystal stucture of the title compound.

The title molecule adopts an *E*-configuration about the central olefinic bonds (Fig.1). The cyclopentanone ring and the furan rings are alomst coplanar. All bond lengths and angles are normal and correspond to those observed in the related substituted cyclopentanone and cyclohexanone analogues reported by Du *et al.* (2007), Sun & Cui (2007) and Wei *et al.* (2008). The crystal packing exhibits weak intermolecular C—H···O hydrogen bonds (Table 1).

### Experimental

Tetrabutylammonium bromide (0.3 mmol) and NaOH (5 mmol) were dissolved in the mixture of water (5 ml) and ethanol (2 ml). The solution was stirred at room temperature for 10 min,followed by added dropwise the mixture of furaldehyde (10 mmol) and cyclopentanone (5 mmol).The mixture was stirred at the temperature of 303 K for 2 h. When the reaction was complete, the residue was filtered. The precipitate was washed by water and recrystallized from ethyl acetate. Analysis calculated for C_15_H_12_O_3_: C 75.00,H 5.00%; found: C 74.90,H 5.03%. Crystals of (I) suitable for single-crystal X-ray analysis were selected after recrystallization.

### Refinement

All H-atoms were initially located in a difference Fourier map and were placed in geometrically idealized positions, with C—H = 0.93 - 0.97 Å, and refined as riding with *U*_iso_(H) = 1.2*U*_eq_(C).

### Crystal data

**Table:**

C_15_H_12_O_3_	*F*(000) = 504
*M**_r_* = 240.25	*D*_x_ = 1.361 Mg m^−^^3^
Monoclinic, *P*2_1_/*c*	Melting point: 405 K
Hall symbol: -P 2ybc	Mo *K*α radiation, λ = 0.71073 Å
*a* = 5.9280 (9) Å	Cell parameters from 461 reflections
*b* = 8.5031 (13) Å	θ = 3.0–18.7°
*c* = 23.280 (3) Å	µ = 0.10 mm^−^^1^
β = 92.139 (3)°	*T* = 298 K
*V* = 1172.6 (3) Å^3^	Block, yellow
*Z* = 4	0.12 × 0.08 × 0.05 mm

### Data collection

**Table:**

Bruker SMART CCD area-detector diffractometer	2076 independent reflections
Radiation source: fine-focus sealed tube	1083 reflections with *I* > 2σ(*I*)
graphite	*R*_int_ = 0.059
φ and ω scans	θ_max_ = 25.1°, θ_min_ = 1.8°
Absorption correction: multi-scan (*SADABS*; Sheldrick, 1996)	*h* = −7→7
*T*_min_ = 0.989, *T*_max_ = 0.995	*k* = −10→9
5793 measured reflections	*l* = −27→19

### Refinement

**Table:**

Refinement on *F*^2^	Secondary atom site location: difference Fourier map
Least-squares matrix: full	Hydrogen site location: inferred from neighbouring sites
*R*[*F*^2^ > 2σ(*F*^2^)] = 0.050	H-atom parameters constrained
*wR*(*F*^2^) = 0.129	*w* = 1/[σ^2^(*F*_o_^2^) + (0.049*P*)^2^] where *P* = (*F*_o_^2^ + 2*F*_c_^2^)/3
*S* = 0.99	(Δ/σ)_max_ < 0.001
2076 reflections	Δρ_max_ = 0.17 e Å^−^^3^
164 parameters	Δρ_min_ = −0.16 e Å^−^^3^
0 restraints	Extinction correction: *SHELXL97* (Sheldrick, 2008), Fc^*^=kFc[1+0.001xFc^2^λ^3^/sin(2θ)]^-1/4^
Primary atom site location: structure-invariant direct methods	Extinction coefficient: 0.015 (2)

### Special details

**Table:**

Geometry. All e.s.d.'s (except the e.s.d. in the dihedral angle between two l.s. planes) are estimated using the full covariance matrix. The cell e.s.d.'s are taken into account individually in the estimation of e.s.d.'s in distances, angles and torsion angles; correlations between e.s.d.'s in cell parameters are only used when they are defined by crystal symmetry. An approximate (isotropic) treatment of cell e.s.d.'s is used for estimating e.s.d.'s involving l.s. planes.
Refinement. Refinement of *F*^2^ against ALL reflections. The weighted *R*-factor *wR* and goodness of fit *S* are based on *F*^2^, conventional *R*-factors *R* are based on *F*, with *F* set to zero for negative *F*^2^. The threshold expression of *F*^2^ > σ(*F*^2^) is used only for calculating *R*-factors(gt) *etc*. and is not relevant to the choice of reflections for refinement. *R*-factors based on *F*^2^ are statistically about twice as large as those based on *F*, and *R*- factors based on ALL data will be even larger.

### Fractional atomic coordinates and isotropic or equivalent isotropic displacement parameters (Å^2^)

**Table:**

	*x*	*y*	*z*	*U*_iso_*/*U*_eq_	
O1	1.2374 (3)	0.2225 (2)	0.62045 (7)	0.0606 (6)	
O2	1.3568 (3)	0.4626 (2)	0.42876 (8)	0.0648 (6)	
O3	0.7570 (3)	−0.1375 (2)	0.73472 (8)	0.0640 (6)	
C1	1.0690 (5)	0.1894 (3)	0.58984 (11)	0.0443 (7)	
C2	1.0293 (4)	0.2376 (3)	0.52998 (10)	0.0416 (7)	
C3	0.8083 (4)	0.1745 (3)	0.50780 (10)	0.0496 (7)	
H3A	0.8296	0.1048	0.4755	0.059*	
H3B	0.7091	0.2597	0.4954	0.059*	
C4	0.7065 (4)	0.0834 (3)	0.55845 (10)	0.0487 (7)	
H4A	0.5655	0.1311	0.5692	0.058*	
H4B	0.6780	−0.0252	0.5478	0.058*	
C5	0.8774 (4)	0.0925 (3)	0.60707 (11)	0.0430 (7)	
C6	1.1804 (4)	0.3239 (3)	0.50282 (11)	0.0476 (7)	
H6	1.3098	0.3508	0.5244	0.057*	
C7	1.1698 (5)	0.3802 (3)	0.44512 (12)	0.0459 (7)	
C8	1.0169 (5)	0.3795 (3)	0.40086 (12)	0.0569 (8)	
H8	0.8755	0.3320	0.4004	0.068*	
C9	1.1100 (6)	0.4640 (3)	0.35521 (12)	0.0652 (9)	
H9	1.0427	0.4831	0.3192	0.078*	
C10	1.3128 (6)	0.5103 (4)	0.37419 (13)	0.0704 (10)	
H10	1.4122	0.5684	0.3526	0.084*	
C11	0.8754 (4)	0.0258 (3)	0.65893 (11)	0.0499 (7)	
H11	1.0011	0.0448	0.6830	0.060*	
C12	0.7047 (5)	−0.0711 (3)	0.68205 (11)	0.0465 (7)	
C13	0.4940 (5)	−0.1134 (3)	0.66577 (12)	0.0579 (8)	
H13	0.4170	−0.0844	0.6319	0.069*	
C14	0.4101 (5)	−0.2101 (4)	0.70957 (13)	0.0657 (9)	
H14	0.2682	−0.2566	0.7101	0.079*	
C15	0.5736 (5)	−0.2212 (4)	0.74968 (13)	0.0642 (9)	
H15	0.5640	−0.2786	0.7835	0.077*	

### Atomic displacement parameters (Å^2^)

**Table:**

	*U*^11^	*U*^22^	*U*^33^	*U*^12^	*U*^13^	*U*^23^
O1	0.0480 (12)	0.0831 (16)	0.0497 (12)	−0.0054 (11)	−0.0089 (9)	0.0018 (10)
O2	0.0632 (13)	0.0725 (15)	0.0587 (14)	−0.0127 (11)	0.0024 (10)	0.0110 (11)
O3	0.0670 (14)	0.0815 (16)	0.0430 (12)	−0.0039 (12)	−0.0042 (10)	0.0100 (10)
C1	0.0404 (16)	0.0489 (18)	0.0437 (17)	0.0062 (14)	0.0016 (13)	−0.0033 (13)
C2	0.0419 (16)	0.0414 (17)	0.0413 (16)	0.0049 (13)	0.0006 (12)	−0.0041 (12)
C3	0.0478 (17)	0.0550 (19)	0.0456 (17)	0.0013 (14)	−0.0021 (13)	−0.0022 (13)
C4	0.0449 (17)	0.0526 (18)	0.0484 (17)	−0.0014 (14)	−0.0006 (13)	−0.0019 (13)
C5	0.0422 (16)	0.0451 (17)	0.0417 (17)	0.0039 (13)	0.0022 (13)	−0.0042 (13)
C6	0.0472 (17)	0.0489 (19)	0.0466 (17)	0.0040 (14)	−0.0018 (13)	−0.0021 (13)
C7	0.0457 (17)	0.0408 (17)	0.0515 (18)	−0.0017 (13)	0.0048 (14)	−0.0021 (13)
C8	0.059 (2)	0.059 (2)	0.0522 (19)	−0.0027 (15)	−0.0028 (16)	−0.0025 (15)
C9	0.090 (3)	0.062 (2)	0.0430 (19)	0.0013 (18)	−0.0044 (17)	0.0024 (16)
C10	0.094 (3)	0.064 (2)	0.054 (2)	−0.0035 (19)	0.0106 (19)	0.0113 (17)
C11	0.0482 (17)	0.058 (2)	0.0435 (17)	0.0025 (14)	−0.0019 (13)	−0.0027 (14)
C12	0.0539 (19)	0.0477 (18)	0.0377 (16)	0.0054 (15)	−0.0009 (13)	0.0013 (13)
C13	0.058 (2)	0.066 (2)	0.0493 (18)	−0.0032 (16)	−0.0052 (15)	0.0082 (15)
C14	0.061 (2)	0.078 (2)	0.058 (2)	−0.0164 (17)	0.0007 (17)	0.0083 (17)
C15	0.072 (2)	0.071 (2)	0.050 (2)	−0.0090 (19)	0.0120 (17)	0.0091 (16)

### Geometric parameters (Å, °)

**Table:**

O1—C1	1.237 (3)	C6—C7	1.425 (3)
O2—C10	1.350 (3)	C6—H6	0.9300
O2—C7	1.377 (3)	C7—C8	1.347 (3)
O3—C15	1.356 (3)	C8—C9	1.412 (4)
O3—C12	1.375 (3)	C8—H8	0.9300
C1—C2	1.463 (3)	C9—C10	1.326 (4)
C1—C5	1.471 (3)	C9—H9	0.9300
C2—C6	1.335 (3)	C10—H10	0.9300
C2—C3	1.490 (3)	C11—C12	1.426 (4)
C3—C4	1.552 (3)	C11—H11	0.9300
C3—H3A	0.9700	C12—C13	1.341 (3)
C3—H3B	0.9700	C13—C14	1.415 (4)
C4—C5	1.493 (3)	C13—H13	0.9300
C4—H4A	0.9700	C14—C15	1.324 (3)
C4—H4B	0.9700	C14—H14	0.9300
C5—C11	1.335 (3)	C15—H15	0.9300
			
C10—O2—C7	106.5 (2)	C8—C7—C6	136.4 (3)
C15—O3—C12	106.8 (2)	O2—C7—C6	115.1 (2)
O1—C1—C2	125.6 (3)	C7—C8—C9	107.6 (3)
O1—C1—C5	125.8 (2)	C7—C8—H8	126.2
C2—C1—C5	108.6 (2)	C9—C8—H8	126.2
C6—C2—C1	121.2 (2)	C10—C9—C8	106.0 (3)
C6—C2—C3	129.1 (2)	C10—C9—H9	127.0
C1—C2—C3	109.7 (2)	C8—C9—H9	127.0
C2—C3—C4	106.2 (2)	C9—C10—O2	111.4 (3)
C2—C3—H3A	110.5	C9—C10—H10	124.3
C4—C3—H3A	110.5	O2—C10—H10	124.3
C2—C3—H3B	110.5	C5—C11—C12	128.1 (2)
C4—C3—H3B	110.5	C5—C11—H11	115.9
H3A—C3—H3B	108.7	C12—C11—H11	115.9
C5—C4—C3	106.2 (2)	C13—C12—O3	108.6 (2)
C5—C4—H4A	110.5	C13—C12—C11	135.6 (3)
C3—C4—H4A	110.5	O3—C12—C11	115.7 (2)
C5—C4—H4B	110.5	C12—C13—C14	107.5 (3)
C3—C4—H4B	110.5	C12—C13—H13	126.2
H4A—C4—H4B	108.7	C14—C13—H13	126.2
C11—C5—C1	121.2 (2)	C15—C14—C13	106.4 (3)
C11—C5—C4	129.4 (2)	C15—C14—H14	126.8
C1—C5—C4	109.3 (2)	C13—C14—H14	126.8
C2—C6—C7	128.6 (2)	C14—C15—O3	110.8 (3)
C2—C6—H6	115.7	C14—C15—H15	124.6
C7—C6—H6	115.7	O3—C15—H15	124.6
C8—C7—O2	108.4 (2)		

### Hydrogen-bond geometry (Å, °)

**Table:**

*D*—H···*A*	*D*—H	H···*A*	*D*···*A*	*D*—H···*A*
C15—H15···O1^i^	0.93	2.49	3.221 (4)	136
C4—H4A···O1^ii^	0.97	2.45	3.393 (3)	165

Symmetry codes: (i) −*x*+2, *y*−1/2, −*z*+3/2; (ii) *x*−1, *y*, *z*.
